# Nano-rectenna powered body-centric nano-networks in the terahertz band

**DOI:** 10.1049/htl.2017.0034

**Published:** 2018-07-13

**Authors:** Zhichao Rong, Mark S. Leeson, Matthew D. Higgins, Yi Lu

**Affiliations:** 1 School of Engineering, University of Warwick, Coventry CV4 7AL, UK; 2 WMG, University of Warwick, Coventry CV4 7AL, UK

**Keywords:** body sensor networks, nanosensors, carbon nanotubes, terahertz wave devices, biomedical telemetry, terahertz band, nano-rectenna powered body-centric nano-networks, wireless body-centric nano-network, nano-sized sensors, healthcare application, nano-batteries, rectifying antennas, carbon nanotubes, wireless information, power transfer, energy bottleneck, ultra-high-speed rectifying diode, external energy source, direct current electricity generation, body-centric nano-networks, nanosensor power sources

## Abstract

A wireless body-centric nano-network consists of various nano-sized sensors with the purpose of healthcare application. One of the main challenges in the network is caused by the very limited power that can be stored in nano-batteries in comparison with the power required to drive the device for communications. Recently, novel rectifying antennas (rectennas) based on carbon nanotubes (CNTs), metal and graphene have been proposed. At the same time, research on simultaneous wireless information and power transfer (SWIPT) schemes has progressed apace. Body-centric nano-networks can overcome their energy bottleneck using these mechanisms. In this Letter, a nano-rectenna energy harvesting model is developed. The energy harvesting is realised by a nano-antenna and an ultra-high-speed rectifying diode combined as a nano-rectenna. This device can be used to power nanosensors using part of the terahertz (THz) information signal without any other system external energy source. The broadband properties of nano-rectennas enable them to generate direct current (DC) electricity from inputs with THz to optical frequencies. The authors calculate the output power generated by the nano-rectenna and compare this with the power required for nanosensors to communicate in the THz band. The calculation and analysis suggest that the nano-rectenna can be a viable approach to provide power for nanosensors in body-centric nano-networks.

## 1 Introduction

**Fig. 1:**
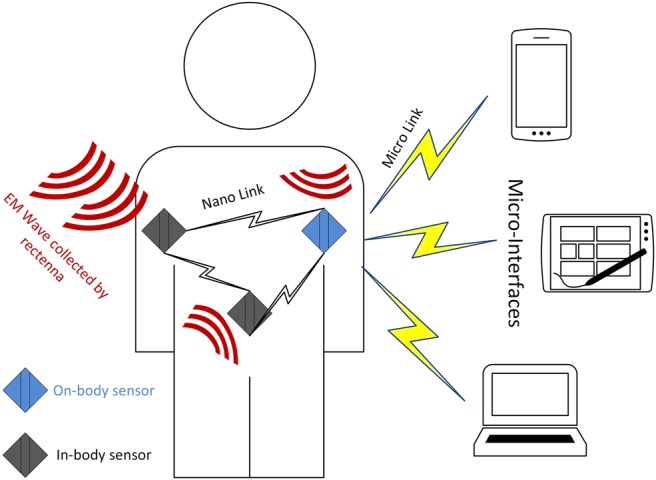
Architecture for wireless body-centric nano-networks. Different nanosensors distributed around and inside the body are used to gather and exchange data. Nano-sensors are equipped with nano-rectennas for harvesting energy and nano-antennas for processing information. A wireless interface among micro devices and nano-sensors are used to collect these data for healthcare centre

Recent developments in nanoscale sensors have led to increasing interest in the interconnection of such devices with established macroscale networks to form the Internet of nano-things (IoNT) [1, 2]. In an IoNT system, a number of nanosensors equipped with fundamental computing and communication abilities can be distributed in the environment for data processing and exchange in the monitoring system. The communication among nanosensors is capable of expanding their abilities to accomplish some more complex tasks [1, 2]. The connected nanosensor network will enable the application of the IoNT in healthcare, military and environmental applications [1]. In the domain of healthcare applications, the purpose is to develop a therapeutic nano-device network which is capable of working either on or inside the human body so as to support immune system monitoring, health monitoring, drug delivery systems and bio-hybrid implants [3]. There are two main approaches for wireless communications at the nanoscale, i.e. molecular and electromagnetic (EM) communications [4]. The latter commonly operates in the terahertz (THz) band (0.1–10 THz) and is a promising technique for supporting data exchange in nanosensor networks for healthcare application or body-centric nano-networks. For the expected size of nanosensors, the frequency radiated by their antennas would ordinarily be in the optical range, resulting in a very large channel attenuation that might render nanoscale wireless communication infeasible. To overcome this limitation, graphene-based antennas have been developed, which are able to resonate in the THz band with sizes of just a few micrometre, at a frequency up to two orders of magnitude lower than a metallic antenna of the same dimensions [5]. A body-centric nano-network, shown in Fig. 1, provides communication among nanosensors either distributed on the body, inside the body or off the body. For example, implantable nano-medical devices, wearable medical sensors and medical information exchange terminals. For the purpose of body-centric nano-networks, communication and information exchange among implantable nanosensors is the most significant as it enables the control and monitoring of the molecular release or flow, biochemical compounds and other important functions inside human body. Information collected from the body area can be then sent via micro-interface to a healthcare centre.

One of the major challenges in body-centric nano-networks is caused by the very limited power storage of a nano-battery. Traditional harvesting mechanisms such as solar cells can convert light waves into a direct current (DC) signal, however, at the nanoscale, the limited size makes the efficiency of solar cells extremely low and they cannot therefore meet the energy demand within body-centric nano-networks. Moreover, sunlight is not available for implant nanosensors and some other parts of the body. Recently, some new energy harvesting methods for powering nanodevices have been proposed [6–9]. For instance, in [6], a piezoelectric-based nanoscale energy harvesting system was experimentally demonstrated. While Jornet and Akyildiz [10, 11] have proposed an energy harvesting system for nano-networks based on a piezoelectric nano-generator. Recently, an ultrasound-driven piezoelectric nano-generator has also been described for powering in-body nanosensors [12]. However, a piezoelectric energy harvesting system is limited to some parts of the body because the power source of this technique is mechanical stress or vibration. For implanted devices in-body, the energy harvesting system requires outside powering such as ultrasound which is not part of THz communication nano-networks. In contrast, wireless power transfer mechanisms based on rectifying antennas (rectennas) offer another promising technique for powering nanodevices in the nano-network [7–9]. Unlike traditional photovoltaic energy harvesters which rely entirely on sunlight, rectennas can operate at THz and microwave frequencies, which enables them to work during the night. Since EM waves carry not only information but also energy [13], nano-rectennas can therefore share the same signal that is used for transporting information within nano-networks. As a result, simultaneous wireless information and power transfer (SWIPT) become a pivotal technique for powering nano-networks and are a promising solution to energy bottlenecks [13–15]. Research on SWIPT has been widely investigated in traditional EM wireless communications [14–17], but there are still no existing studies in the area of THz band communication at the nanoscale. In this Letter, we focus on the design of nano-rectennas which will be the key elements of SWIPT systems in the THz band. A major advantage of the technique is that the proposed nano-rectennas are able to convert an EM signal into a DC current without any external system power source. Moreover, the achievable energy conversion efficiency for a rectenna in principle is very high. For example, efficiencies of around 85% have been reported in [18].

**Fig. 2:**
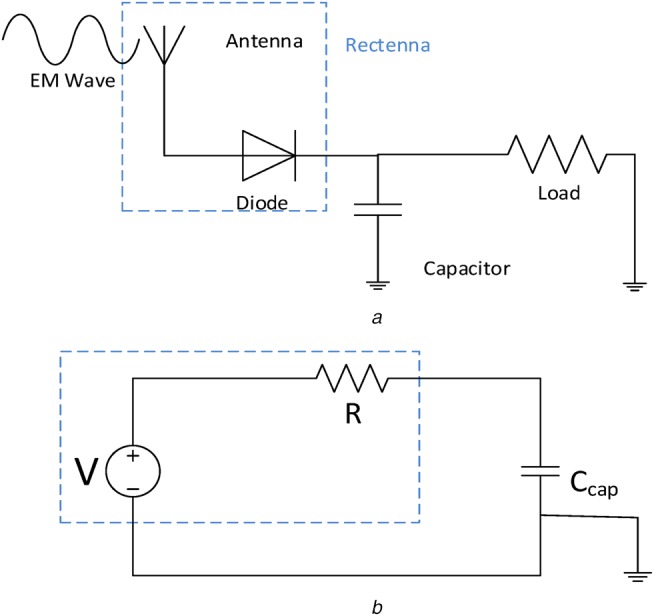
Schematic diagram of a rectenna *a* Antenna and diode coupled as a rectenna *b* Simplified system equivalent RC circuit

A rectenna, shown schematically in Fig. 2*a*, is a combination of an antenna and a rectifying device, usually a diode. For the purpose of energy harvesting in nano-networks, the EM waves are received by a nano-antenna and then coupled to a rectifier to complete the rectenna configuration. Therefore, the core components of a nano-rectenna are a high-speed rectifier (diode) and a nanoscale antenna, which can be used for harvesting energy from THz and higher frequencies. As nano-sized antennas operate in the THz band, their associated rectifying diodes need a fast response so that they can react appropriately to the incoming THz signal and deliver a DC signal. The nano-antenna collects high-frequency freely propagating EM which it converts into AC current to the ultrafast diode, which then converts this current to DC. In [7–9], different kinds of nano-rectenna have been demonstrated experimentally, in [7], a bowtie dipole gold nano-antenna with a metal-insulator-metal diode has been fabricated and measured. The rectenna, which operates around 28.3 THz and higher frequencies, can harvest energy from the THz signal or from waste energy in the ambient environment. The device comprised an insulator copper oxide (CuO) sandwiched between gold (Au) and copper (Cu) to make the Au/CuO/Cu MIM structure and had a responsivity at zero bias of 5 AW^−1^. A carbon nanotube (CNT)-based rectenna has been proposed in [8] which consisted of millions of CNTs operating as nano-antennas with their tips fabricated with insulator-metal to behave as diodes. The CNT rectenna showed great potential for wireless EM powering body-centric nanodevice applications.

In this Letter, a novel application of an energy harvesting method for nanosensors based on a nano-rectenna is proposed for healthcare nano-networks. The available energy that a nano-rectenna can harvest at a nanosensor is calculated and the potential output power is computed. For both the CNT rectenna array and the Au/CuO/Cu rectenna array, we present analytical expressions for the time taken to charge a super-capacitor and compare the performance with the existing system (piezoelectric with ultrasound). Our results show that a 25-element Au/CuO/Cu rectenna array provides the best performance and is able to meet the energy requirement in a body-centric nano-network. Our findings show that the proposed system offers a considerable advantage over the existing system.

This Letter is organised as follows: Section 2 introduces the energy harvesting system with different nano-rectennas. The following section demonstrates the power output of energy harvesting system and the corresponding performance; numerical results for comparison among different systems are also presented. Finally, Section 4 concludes the key findings and the future work of the Letter.

## 2 Energy harvesting using nano-rectenna

In this section, we study the nano-network energy harvesting system using a CNT nano-rectenna and an Au/CuO/Cu nano-rectenna. As stated above, since traditional energy harvesting schemes are not available for body-centric nano-networks a rectenna-based scheme is promising. If we treat the rectenna as a nano-generator as shown in Fig. 2*b*, it consists of the nano-rectenna, e.g. a nanotube rectenna and an ultra-nanocapacitor. The rectenna is represented by its series resistance *R* and output voltage *V* (from an EM wave), which is rectified by the diode to supply a DC charging current to an ultra-nanocapacitor $C_{cap}$.

**Fig. 3:**
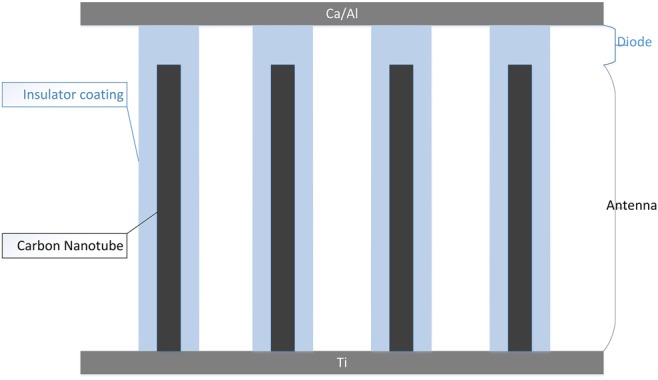
Schematic diagram of the CNT rectenna. Insulator-coated CNTs are vertically aligned at a density of 10^10^ cm^−2^ on a coated metal (Ti) substrate, and with their tips capped with Ca/Al. CNTs behave as antennas which collect EM waves to the tip areas (which act as diodes), where the waves are converted to direct current

For example in [8], the CNT rectenna is as shown in Fig. 3, and the CNTs behave as antennas with their small tip areas acting as rectifying diodes. When the CNTs absorb EM radiation, a DC current will be generated after rectification by the tip area. This converted current is used to charge a capacitor [8]. The conversion process continues using the THz signal within the system and ambient-free EM so the energy source of such a nano-rectenna generator needs no other specific external power source.

**Fig. 4:**
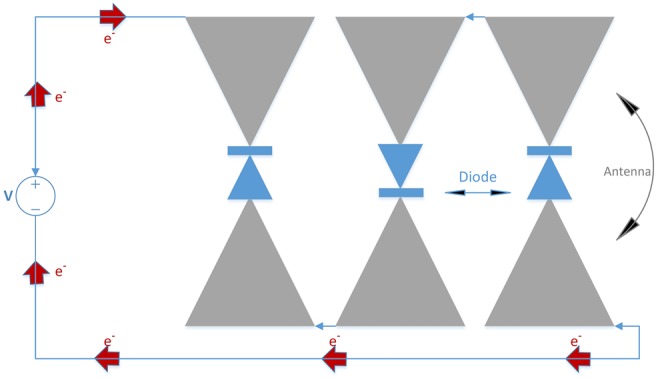
Schematic diagram of bowtie nano-rectenna array

In [7, 9, 19, 20], bowtie dipole nano-rectennas have been proposed and have the form shown in Fig. 4; they are fabricated in gold with lengths of ∼5–6 μm with two 2–3 μm triangular sections. The antenna thickness is 100 nm, and the nano-diodes, made from graphene [9, 19] or MIM [7], are located in the middle of the bowtie antenna gap area, producing the rectenna action. A series of these rectennas can be connected to form a nano-rectenna array shown in Fig. 4. The bowtie dipole antenna receives EM radiation and converts the signal to AC current flow to the nano-diode. The diode then rectifies the AC electricity to DC electricity. When connected to an ultra nanocapacitor as is shown in Fig. 2, the rectified DC electricity can be harvested and used by nanosensors.

For the THz frequency range, the nano-rectenna can be analysed using the classical formulas for macroscale devices [21]. The performance of a nano-rectenna is mainly determined by three characteristics, the first and the most important one is responsivity which is defined as the direct current generated by the induced EM radiation power over the rectenna. It represents the amount of DC current that can be induced for a given input AC EM wave power and can be calculated as [22]

(1) $$\beta = \displaystyle{1 \over 2}\displaystyle{{{I}^{\prime \prime}\lpar V_{{\rm bias}}\rpar } \over {{I}^{\prime}\lpar V_{{\rm bias}}\rpar }}\eqno\lpar 1\rpar $$

which is the ratio of the second to the first derivative of the current. For the purpose of energy harvesting, the bias voltage will be set to zero.

The diode resistance is the second important characteristic which determines the performance of the rectenna. Therefore, resistance matching between the antenna and the diode is important. For instance, in [7], the reported rectenna has an antenna resistance of 100 Ω and a diode resistance of 500 Ω which results in a relatively good match.

Thirdly, the diode's cut-off frequency is another important characteristic. The rectenna EM wave absorption efficiency is limited by this cut-off frequency, all frequencies lower than this frequency can be harvested. Moreover, for a nano-antenna, the required frequency is in the THz range. The cut-off frequency of a rectenna can be calculated using:

(2) $$f_{\rm c} = \displaystyle{1 \over {2\pi R_{{\rm rec}}C_{\rm D}}}\eqno\lpar 2\rpar $$

where *R*_rec_ is the rectenna's equivalent resistance, *C*_D_ is the capacitance of the diode (determined from the standard expression in terms of the device permittivity and physical dimensions).

The DC current that generated by the rectenna can be calculated from:

(3) $$I = \left[{I\lpar V_{{\rm bias}}\rpar + \displaystyle{1 \over 2}\displaystyle{\beta \over {R_{\rm d}}}V_{{\rm ac}}^2 } \right]\eqno\lpar 3\rpar $$

where *V*_ac_ is the input AC voltage and *R*_d_ is the rectifier differential resistance.

Otherwise, the current generated by the rectenna can also be calculated based on the input power from:

(4) $$I = P_{{\rm in}}A_{{\rm eff}}\eta _{\rm a}\beta \eta _{\rm c}\eqno\lpar 4\rpar $$

where *P*_in_ is the input EM wave power, *A*_eff_ is the effective area of the antenna, *η*_a_ is the absorption efficiency of the antenna and *η*_c_ is the rectenna coupling efficiency

(5) $$\eta _{\rm C} = 4\displaystyle{{R_{\rm a}R_{\rm d}/{\left({R_{\rm a} + R_{\rm d}} \right)}^2} \over {1 + {\left({2\pi f\lpar R_{\rm a}R_{\rm d}/\lpar R_{\rm a} + R_{\rm d}\rpar \rpar C} \right)}^2}}\eqno\lpar 5\rpar $$

where *f* is the frequency of the radiation received by the antenna and *R*_a_ is the resistance of the antenna.

The DC voltage generated from the rectenna can be calculated as

(6) $$V_{\rm D} = - \displaystyle{1 \over 2}\beta V_{{\rm opt}}^2 \eqno\lpar 6\rpar $$

where *V*_opt_ is the AC output voltage of the antenna.

Therefore, the output power of the rectenna can be calculated from

(7) $$P_{{\rm out}} = - \displaystyle{{\beta ^2V_{{\rm opt}}^4 } \over {16R_{\rm d}}}\eqno\lpar 7\rpar $$

## 3 Output power analysis and comparisons

**Fig. 5:**
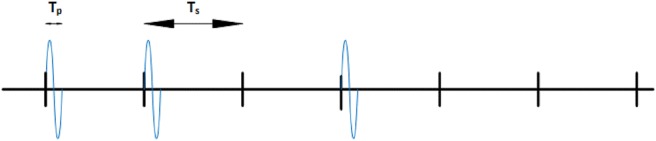
Transmitting the sequence ‘110100’ with a pulse duration of 100 fs and symbol duration of 100 ps

According to the results reported in [8], the output voltage generated by the CNT rectenna is of the order of tens of millivolts. For instance, the output power using a 1064 nm input EM wave can be calculated based on the results obtained from [8], which are *V*_opt_ = 68 mV, *β* = 0.4 AW^−1^, and *R*_d_ = 80Ω, permitting calculation of the output power of the rectenna from (7) as 2.67 nW. In accordance with [3, 23], a femtosecond pulse-based channel access scheme will be applied to the nano-network, shown in Fig. 5, those digits ‘1’ are transmitted using 100 fs (i.e. *T*_p_ = 100 fs) long pulse while digits ‘0’ are transmitted as silence. For example, in Fig. 5, the sequence ‘110100’ is transmitted. According to [14], the required peak pulse power is reported to be 1–10 μW (i.e. 10^−18^ J of energy). As the separation time among adjacent bits (symbol duration) is 1000 times the pulse duration (*T*_s_ = 100ps), the average power will be brought back to the nW level [12]. Thus, the output power of the CNT rectenna is able to satisfy the power requirement of the system. In [7], the reported rectenna performs with a responsivity of 5 AW^−1^ and resistance of 500 Ω at zero bias. The contact area of the diode is 0.0045 μm^2^, with a 7 nm thickness and relative dielectric constant of CuO is 18.1. Using these values gives the diode's capacitance as 10^−16^ F. The rectenna coupling efficiency can, therefore, be calculated from (3) to be 17.4% operating with a 28.3 THz EM input. As is reported in [24], the effective area of the antenna is 37.5 µm^2^ and the absorption efficiency is 37%. When 49 mW mm^−2^ power input to the rectenna, according to (4), the output DC current is 0.47 µA and hence the calculated power output of this single nano-rectenna is 0.11 nW.

**Fig. 6:**
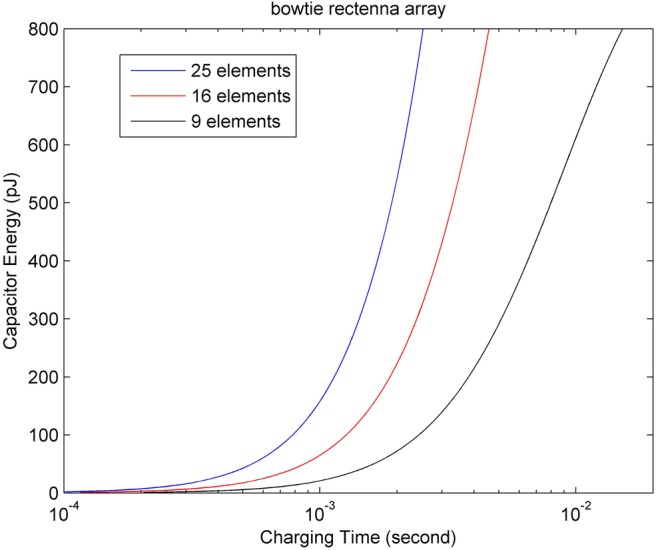
Energy production ability compassion among different number of array elements for bowtie rectenna

According to the previous results computed for the CNT rectenna, it can generate a power output of nearly 3 nW with CNT area of 5 × 10^−5^ cm^2^ or 5000 μm^2^ which contains about 10^8^ nanotube rectennas. While the single bowtie rectenna is 10.6 μm long with a 50° bow angle and the size is reported to be around 37.5 μm^2^. However, the target size of the implantable nanosensors is expected to be 10–1000 μm^2^, hence, the CNT rectenna is better for use in powering on body devices whilst the bowtie rectenna can be used for implantable nanodevices. As the power output of a single bowtie rectenna is 0.11 nW, if we use an array of these rectennas the required power and size can be satisfied. More elements connected in series can increase the production of current and power, Fig. 6 illustrates the energy production ability for different number of array elements. We assume that the rectenna array consists of 25 elements, which are all perfectly coupled to give a maximum output power of 2.75 nW. As shown in Fig. 1, the rectenna is treated as a generator with an ideal power source *V* and resistance *R*. The charging voltage to the ultra-capacitor is

(8) $$V_{\rm c} = V\lpar 1 - {\rm e}^{ - \lpar t/{\rm R}{\rm C}_{{\rm cap}}\rpar }\rpar \eqno\lpar 8\rpar $$

The energy that is stored in the ultra-nanocapacitor is then calculated as

(9) $$E = \displaystyle{1 \over 2}C_{{\rm cap}}V_{\rm c}^2 \eqno\lpar 9\rpar $$

**Fig. 7:**
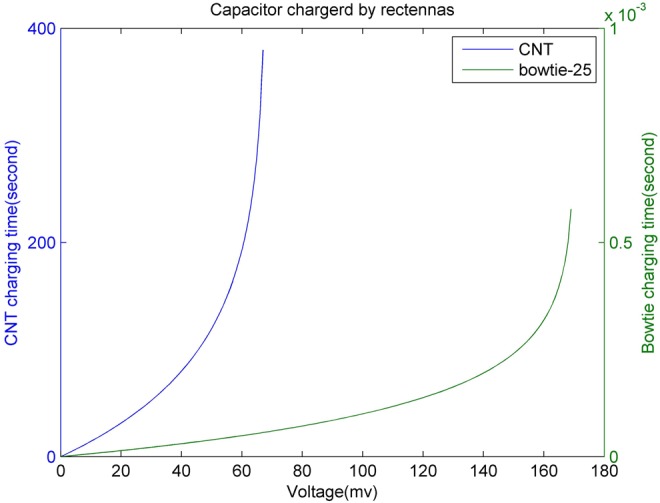
Time taken for CNT rectenna and 25-element bowtie rectenna array charging the ultra-nano-capacitor, the bowtie rectenna array produce a higher voltage output

**Fig. 8:**
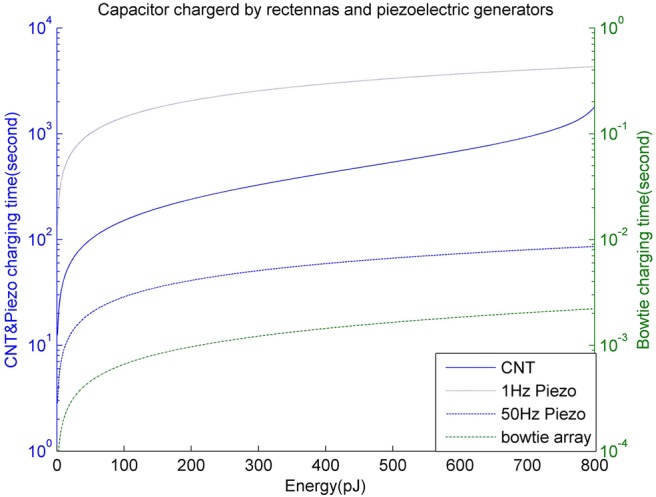
Time taken for CNT rectenna and 25-element bowtie rectenna array and piezoelectric nano-generators charging the ultra-nano-capacitor, the bowtie rectenna array is the most efficient while piezoelectric nano-generator supply the highest output voltage

In [10], a 9 nF ultra-nanocapacitor (with the areal capacitance of 0.9 mF·cm^−2^ and a size of 1000 μm^2^) was used and the resulting maximum capacity from (9) is about 800 pJ. The harvesting system based on piezoelectric technique takes 50 s to charge a 9 nF nanocapacitor for 50 Hz external vibrations (i.e. ultrasound) and some 42 min using 1 Hz external vibrations [10–12]. For a CNT rectenna device, the maximum output voltage reported is 68 mV and for a 25-element bowtie rectenna array it is 170 mV. Therefore, according to (9), bowtie rectenna array delivers more charge than the CNT rectenna. As shown in Fig. 7, when both these rectenna devices are used to charge the same ultra-nanocapacitor (9 nF), it is apparent that the CNT rectenna takes more time (over 6 min) because of its very high junction resistance. While for the bowtie rectenna, the resistance is comparably very small thus it just takes about 6 ms to supply more energy for the capacitor. In [25], a novel 3D structure ultra-capacitor has been demonstrated, which supports an areal capacitance of over 100 mF·cm^−2^, for instance, we used a 350 μm^2^-sized capacitor to compare the charging time for different energy harvesting devices, i.e. a CNT rectenna, a 25-element bowtie array rectenna and a piezoelectric nano-generator. According to the results shown in Fig. 8, it takes 30 min to charge the capacitor reaching 800 pJ energy for a CNT rectenna, and 2.2 s, 86 s, 71.7 min for a 25-element bowtie array rectenna, a piezoelectric nano-generator with 50 and 1 Hz external vibration, respectively. Therefore, the smallest-sized 25-element bowtie rectenna is the most efficient and moreover, this rectenna does not need any external system energy source while producing DC directly from EM signals of broadband frequencies as well as the CNT rectenna. While a piezoelectric generator requires external system driving power (ultrasound) which is a big drawback in contrast to nano-rectennas. However, the piezoelectric nano-generator supplies the highest output voltage, i.e. 0.42 V, which enables the application of more requirements for nanosensors. Furthermore, in contrast to an RF rectenna where its antenna and rectifier work independently, the two components of both CNT rectenna and bowtie rectenna are fabricated compactly which can reduce the propagation loss and achieve a good conversion efficiency [7, 8]. Table 1 shows the general properties of different schemes. Note that the total size of a piezoelectric nano-generator is comprised of an area of 1000 μm^2^ nanowires, 1000 μm^2^ ultra-capacitor and space for wiring.

**Table 1:** General properties of different nanoscale energy harvesting schemes

Schemes	Size, μm^2^	Output voltage	Energy source
Piezo (50 Hz)	>2000	0.42 V	ultrasound (system external)
Piezo (1 Hz)	>2000	0.42 V	ultrasound (system external)
CNT array	∼5000	68 mV	THz to optical
Bowtie array (25 elements)	∼1000	170 mV	∼28 THz

## 4 Conclusion

In this Letter, the options for energy harvesting systems based on nano-rectennas for wireless body-centric nano-networks have been investigated. The energy harvesting scheme for body-centric nano-networks is developed based on nanoscale rectennas which act as generators in the system. Along with the continuing advancement of the SWIPT technique, the pioneering CNT array rectenna and the bowtie array nano-rectenna open the door for the wireless powering of nanosensors. Since a nano-rectenna is able to power nanosensors without any external system source and its broadband property enables rectenna to be a very efficient and promising way to power implanted and body area nanodevices. This Letter has briefly analysed the new energy harvesting mechanism and its application in nanosensor network. The CNT array rectenna can successfully supply the required power of the wireless body-centric area at around 27.5 nW. Moreover, the bowtie array rectenna is of a much smaller size but provides similar power. In this Letter, we have also compared the two nano-rectennas with a piezoelectric nano-generator. Although nano-rectennas cannot provide as high a voltage when compared with a piezoelectric nano-generator, a bowtie nano-rectenna array is much more efficient while producing DC directly from the THz signal within the system and ambient EM signal without any other system external power source. Finally, our overall aim was to examine the options for nanoscale energy harvesting that could be used in healthcare. Thus we have employed relatively simple models for the devices to establish their underlying features. We therefore acknowledge that there is work to be done in the development of simulation models for the rectennas.

## 5 Funding and declaration of interests

None declared.
